# Antitumor effect of FP3 in a breast cancer xenograft model

**DOI:** 10.3892/etm.2012.773

**Published:** 2012-10-26

**Authors:** HUANRONG LAN, LINGZHI ZHENG, KETAO JIN, LISONG TENG

**Affiliations:** 1 Departments of Plastic Surgery; 2 Gynecology and Obstetrics and; 3 Surgical Oncology, Taizhou Hospital, Wenzhou Medical College, Linhai, Zhejiang 317000;; 4 Department of Surgical Oncology, First Affiliated Hospital, College of Medicine, Zhejiang University, Hangzhou, Zhejiang 310003, P.R. China

**Keywords:** FP3, vascular endothelial growth factor, breast cancer, antitumor effect, antiangiogenic effect

## Abstract

FP3 is a novel vascular endothelial growth factor (VEGF) blocker proposed to have antiangiogenic properties. Previous studies revealed that FP3 is a new promising agent for treating human choroidal neovascularization (CNV)-associated age-associated macular degeneration (AMD) and has an inhibitory effect on VEGF-mediated proliferation and migration of human umbilical vein endothelial cells and VEGF-mediated vessel sprouting of the rat aortic ring *in vitro*. Previous studies have also revealed that FP3 has antitumor effects and antiangiogenic effects in a non-small cell lung cancer cell line (A549), as well as in patient-derived tumor tissue xenograft models of gastric cancer and colon carcinoma with lymphatic and hepatic metastases in nude mice. In the present study, the antitumor effect of FP3 in an MDA-MB-231 breast cancer xenograft model was investigated. Treatment with FP3 for 3 weeks significantly suppressed xenograft growth and this inhibition was associated with a significant decrease in angiogenesis and direct inhibition of tumor cells. The results of the present study indicate that FP3 inhibits breast cancer tumor growth via the indirect inhibition of angiogenesis as well as a direct effect on tumor cells.

## Introduction

Angiogenesis is the process of new blood vessel formation. It is an important process in the growth of malignant tumors as solid tumors must develop an angiogenic phenotype which promotes the establishment of an expanding vascular network for the delivery of oxygen and other nutrients (1). The predominant regulator of tumor angiogenesis is vascular endothelial growth factor (VEGF) (2,3) which is the only angiogenic factor known to be present throughout the entire tumor lifecycle (2,4). VEGF promotes endothelial cell proliferation, migration and survival in support of tumor angiogenesis. In addition, VEGF is a potent stimulator of vessel permeability, having originally been recognized for its function as a vascular permeability factor (5,6). Due to its fundamental role in tumor angiogenesis, VEGF serves as a logical target for antiangiogenic cancer therapy. Breast cancer is the most common neoplasm in women (7). Tumor angiogenesis is essential for the growth and spread of breast cancer cells. There are at least 6 different angiogenic growth factors associated with tumor angiogenesis in breast cancer. The major mediator of tumor angiogenesis is VEGF (8). VEGF expression is increased in a number of tumor types, including breast cancer (9). Overexpression of VEGF is associated with a poor prognosis for patients with breast cancer (10). In addition to its prognostic role, VEGF is also a validated target in the treatment of this disease. Recently, various antiangiogenic agents have shown efficacy in the treatment of breast cancer (11,12).

FP3 (also known as KH902 or KH903) is an engineered protein which contains the extracellular domain 2 of VEGF receptor 1 (Flt-1) and extracellular domains 3 and 4 of VEGF receptor 2 (Flk-1, KDR) fused to the Fc region of human immunoglobulin G1 (11,13). Previous studies revealed that FP3 has promise as a local antiangiogenic treatment for human choroidal neovascularization (CNV)-associated age-associated macular degeneration (AMD) (11,14–16). In subsequent studies, it has been demonstrated that FP3 has an inhibitory effect on the VEGF-mediated proliferation and migration of human umbilical vein endothelial cells and VEGF-mediated vessel sprouting of the rat aortic ring *in vitro*(13). Previous studies also revealed that FP3 has antitumor effects and anti-angiogenic effects in a non-small cell lung cancer cell line (A549) (13) and patient-derived tumor tissue xenograft models of gastric cancer (17), as well as in colon carcinoma with lymphatic and hepatic metastases in nude mice (18).

It is unknown whether FP3 has an antitumor effect in breast cancer and what the mechanism behind the potential effect of FP3 would be. For this purpose, the present study was designed to evaluate the potential antitumor effects of FP3 in a breast cancer cell line subcutaneous xenograft model in nude mice and assess the antiangiogenenic effects of FP3 in this model.

## Materials and methods

### Materials

The MDA-MB-231 human breast cancer cells were purchased from American Type Culture Collection (ATCC, Manassas, VA, USA). RPMI-1640 medium, fetal bovine serum (FBS), penicillin and streptomycin were purchased from Gibco (Grand Island, NY, USA). Methanol, acetic acid, crystal violet and hydrogen peroxide were purchased from Sigma (St. Louis, MO, USA). The antibody against platelet endothelial cell adhesion molecule-1 (PECAM-1, CD31; rat monoclonal, clone MEC 13.3) was purchased from BD Pharmingen (San Diego, CA, USA). Fluorescent (Cy3-conjugated) secondary antibody (goat anti-rat) was purchased from Jackson ImmunoResearch (West Grove, PA, USA). Bevacizumab (Avastin) was purchased from Roche, Inc. (Roche, Nutley, NJ, USA). FP3 was provided as a gift by Kanghong Biotechnology Inc. (Kanghong, Chengdu, China).

### Animals

Female BALB/c nude mice (4–6 weeks old) purchased from Slaccas Laboratory Animal Co. (Shanghai, China) were housed in a barrier facility and acclimated to 12-h light-dark cycles for ≥3 days prior to use. The use of experimental animals adhered to the ‘Principles of Laboratory Animal Care’ (NIH publication #85-23, revised in 1985). All experiments were approved by the Institutional Animal Care and Use Committee of Zhejiang University (approval ID: SYXK(ZHE)2005-0072).

### Cell culture

MDA-MB-231, a breast cancer cell line, was maintained in RPMI-1640 medium supplemented with 10% FBS, 200 IU/ml penicillin and 200 *μ*g/ml streptomycin. The culture medium was replaced every other day. After reaching confluence, the cells were subcultured.

### In vitro MDA-MB-231 cell clonogenic assay

MDA-MB-231 cells were cultured in RPMI-1640 with 10% FBS. Cells in the exponential growth phase were trypsinized, washed and counted. The cells were seeded in triplicate at a density of 2,500 cells per 100-mm dish containing 10 ml complete medium. Cells were treated 2 h after seeding with FP3 (20, 50 or 100 *μ*g/ml) or bevacizumab (50 *μ*g/ml). The control plates received medium only. After 10 days of incubation at 37°C, the medium was drained and colonies were rinsed, fixed with a mixture of methanol and acetic acid (10:1) and stained with 1% crystal violet. The colonies containing >50 cells were counted.

### Mice tumor xenografts

For inoculation, ∼1.0×10^7^ MDA-MB-231 cells in 0.2 ml serum-free RPMI-1640 were injected subcutaneously into the right flank of 32 female athymic nude mice. The mice developed visible tumors within 3 weeks of the inoculation. The tumors were allowed to grow to >60 mm^3^ prior to imaging. Subsequently, the mice were divided into 4 treatment groups containing 8 mice each and the treatments were initiated. An 8-mouse group was used as an implantation and normal saline (NS)-treated negative control.

### Mouse drug treatments and tumor growth regression assay

Following the tumor cell implantation and within 2 weeks of inoculation, the mice received 200 *μ*l vehicle (NS), FP3 (2, 6 or 18 mg/kg) or bevacizumab (Avastin; 6 mg/kg) intravenously. The mice were treated with the drugs from 21 days after implantation at the indicated doses twice per week (n=8 for each dose) for 3 weeks. The animals were then sacrificed and the tumors were measured *ex vivo* with calipers (tumor volume = length × width^2^ /2).

### Immunohistochemical staining

Formalin-fixed, paraffin-embedded 5-*μ*m thick tumor sections were analyzed by immunohistochemical analysis according to the previously described method (18). The sections were baked, deparaffinized in xylene and rehydrated. Antigen retrieval was performed with citrate buffer (pH 6.0) in a microwave at 98°C for 5 min. Following a PBS wash, endogenous peroxidases were quenched in 3% hydrogen peroxide for 20 min. The slides were then blocked with 1% normal goat serum for 20 min at room temperature and incubated with rat anti-mouse PECAM-1 (CD31) polyclonal antibody at a 1:100 dilution. The slides were incubated overnight at 4°C. The following day, the slides were washed several times with PBS and the specimens were incubated for 1 h at room temperature with fluorescent (Cy3-conjugated) secondary antibody (goat anti-rat) diluted (1:200) in PBS. Specimens were rinsed again with PBS and mounted in Vectashield (Vector Laboratories, Burlingame, CA, USA). The tissue sections were examined and digitally photographed using a Zeiss Axiophot fluorescence microscope (Carl Zeiss, Thornwood, NY, USA) equipped with single, dual and triple fluorescence filters and a low-light, externally cooled, three-chip charge-coupled device (CCD) camera (480×640 pixel RGB-color images, CoolCam; SciMeasure Analytical Systems, Atlanta, GA, USA) and saved as TIFF files.

### Statistical analysis

Data are presented as the mean ± SEM and were analyzed using SPSS 16.0 software (SPSS, Inc., Chicago, IL, USA). Differences among the means of the groups were determined using one-way ANOVA. P<0.05 was considered to indicate a statistically significant difference.

## Results

### FP3 significantly inhibits MDA-MB-231 cell proliferation in vitro and blocks tumor growth in vivo

**Figure 1:**
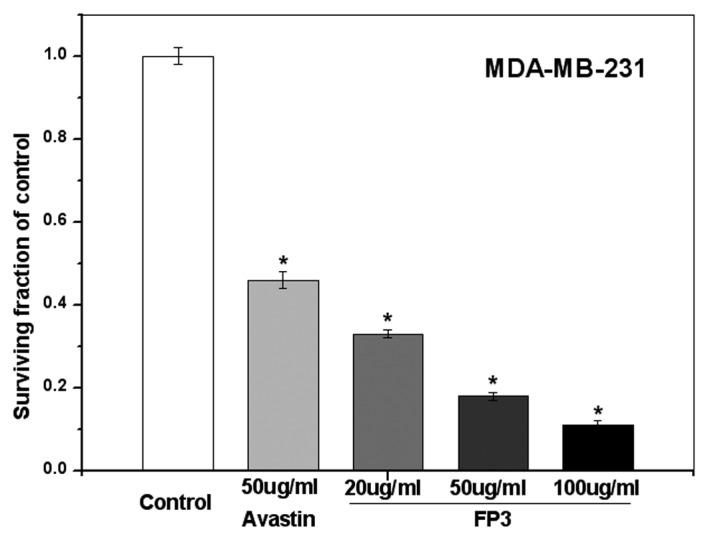
Antiproliferative effect of FP3 on MDA-MB-231 cells *in vitro*. Clonogenicity of MDA-MB-231 cells was reduced by 77, 82, and 89% following treatment with 20, 50 amd 100 *μ*g/ml FP3, respectively, for 10 days at 37°C. Cell proliferation was normalized to NS controls. Data are the mean ± SEM from 3 independent experiments. ^*^ p<0.01, vs. NS control. NS, normal saline.

The antiproliferative effect of FP3 on MDA-MB-231 cells *in vitro* was evaluated. The results demonstrated that FP3 directly inhibited the tumor cells. FP3 reduced MDA-MB-231 cell colony formation by >77%. (Fig. 1)

**Figure 2:**
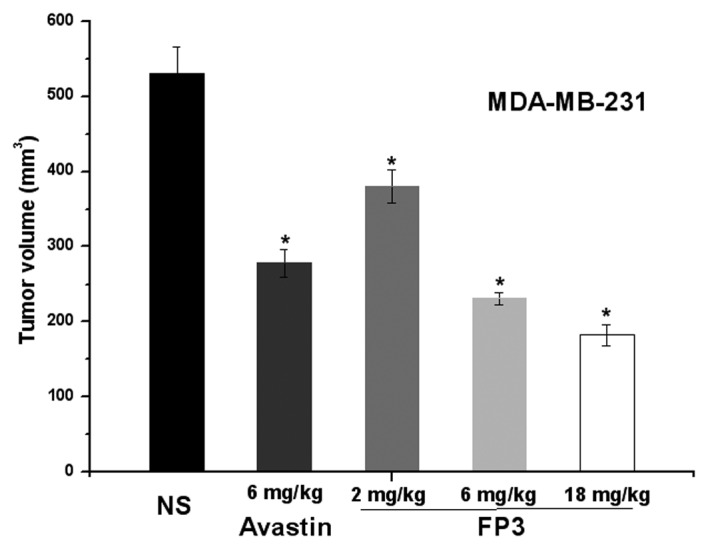
FP3 significantly inhibits the subcutaneous growth of implanted MDA-MB-231 tumors. FP3 substantially blocked the growth of the subcutaneously implanted tumor when administered at the indicated doses twice weekly for 3 weeks. Error bars represent the standard error of mean; n=8 mice/treatment group. Data shown are the mean ± SEM. The differences between control tumor volumes, Avastin-treated and FP3-treated tumor volumes were analyzed using one-way ANOVA. ^*^p<0.001, vs. NS control. NS, normal saline.

To begin to evaluate FP3 as an anticancer therapeutic agent for breast cancer and to compare it with other effective agents targeting the VEGF pathway, its ability to block the growth of a breast cancer cell line, MDA-MB-231, in a mouse subcutaneous tumor model was evaluated. Following implantation, tumor cells were allowed to grow for 3 weeks and formed large retroperitoneal tumors >60 mm^3^. Injections of FP3 (2, 6 and 18 mg/kg body weight), bevacizumab (6 mg/kg body weight) or NS were then administered intravenously, biweekly for 3 weeks, after which the animals were sacrificed and the tumors excised and measured. FP3 significantly inhibited the growth of the tumor xenografts (Fig. 2).

### FP3 results in decreased vasculature of tumors

**Figure 3:**
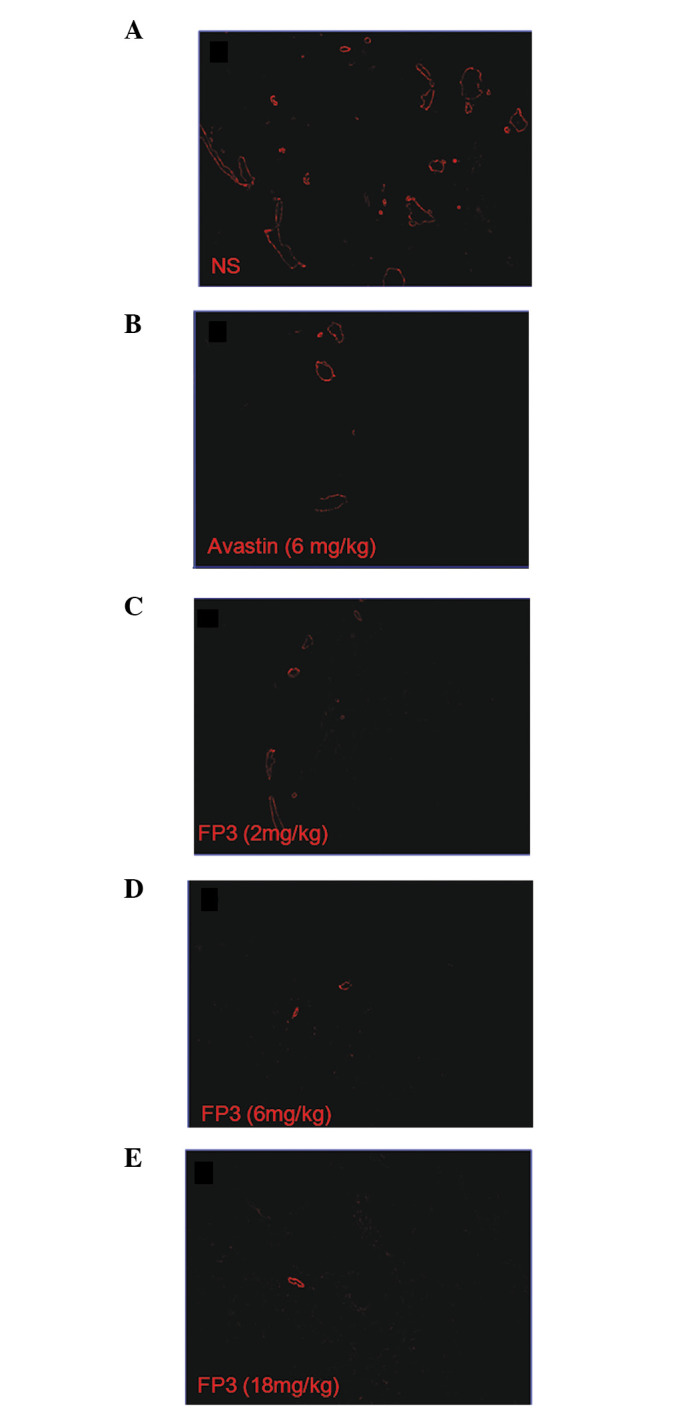
FP3 decreased vascular structure. Vasculature was examined by angiography with immunostaining for endothelial cells (using anti-PECAM-1 antibody; bar=100 *μ*m). NS, normal saline; PECAM-1, platelet endothelial cell adhesion molecule-1.

To evaluate the effects of FP3 on tumor-associated angiogenesis, the tumor xenografts were sectioned and immune-stained with an anti-body to PECAM-1 so that the vasculature was visualized. This analysis revealed that the higher doses of FP3 almost completely blocked tumor-associated angiogenesis, with the stunted tumors being largely avascular (Fig. 3D and E). The lowest dose of FP3 (2 mg/kg) was not as effective at inhibiting tumor growth compared to a higher dose (6 or 18 mg/kg). However, the lowest dose of FP3 (2 mg/kg) appeared to be as effective as the higher doses (6 or 18 mg/kg) at blocking tumor-associated angiogenesis (Fig. 3C–E). In contrast to the FP3-treated tumors, the control tumors in the vehicle-treated mice were not only much larger but also had a high vascular density (Fig. 3A).

## Discussion

Tumor vessels are considered to be dynamic in terms of the formation of new vessels or angiogenesis. Tumors acquire their vasculature by endothelial cell sprouting, co-option of pre-existing vessels, intussusceptive microvascular growth, postnatal vasculogenesis, glomeruloid angiogenesis or vasculogenic mimicry. It should be emphasized that in the majority of cases, these mechanisms are interlinked, participating simultaneously in physiological and pathological angiogenesis (11). VEGF promotes certain or all of these processes, rendering VEGF a rational target for antiangiogenic drug development (19). Since anti-VEGF approaches act by blocking tumor-associated angiogenesis, which appears to be widely required by numerous different types of tumors, these approaches may prove to be generally useful against a wide variety of cancer types (20).

VEGF expression is increased in a number of tumor types, including breast cancer (9). VEGF-A is highly expressed in numerous tumors of the lung, brain and gastrointestinal and urogenital tracts, as well as *in situ* and invasive breast cancer (21). There is a positive correlation between VEGF levels and poor clinical outcomes, including patient survival (10). Anti-VEGF treatment inhibits the growth of human breast tumor xenografts in animals (22).

FP3 is a humanized fusion protein which combines ligand binding elements taken from the extracellular domains of VEGF receptors 1 and 2 and the Fc portion of IgG1 and is designed to bind to all forms of VEGF-A (13). In order to further substantiate the antitumor and anti-angiogenesis effects of FP3 in breast cancer, an MDA-MB-231 subcutaneous xenograft model in nude mice was used. The results of the study showed that, in the MDA-MB-231 human breast cancer xenograft model, FP3 effectively inhibited tumor growth (Fig. 2). Treatment with FP3 also resulted in stunted and almost completely avascular tumors (Fig. 3). The antitumor activity of FP3 is most likely mediated by the inhibition of angiogenesis since the microvessel density values in FP3-treated tumors were significantly decreased. The fact that FP3 resembled the well-defined angiogesis inhibitor bevacizumab with regard to tumor growth and microvessel density measurements, is an additional indication of the antiangiogenic activity of FP3.

Whether FP3 has a direct cell-killing or inhibitory effect in MDA-MB-231 cells *in vitro* was investigated using a clonogenic assay. FP3 was identified to have a direct cell-killing effect on MDA-MB-231 cells (Fig. 1). These results indicated that the inhibitory effect of FP3 on the MDA-MB-231 tumor xenograft model growth may partially result from the inhibition of the tumor cells.

The results of the present study reveal that FP3 has an excellent antitumor effect against breast cancer xenografts and indicate that it may have potential as an effective antiangiogenic agent in the treatment of breast cancer.
